# Cadmium Enrichment Characteristics in Different *Oratosquilla oratoria* Tissues During Various Gonadal Development Stages from Shanghai and Its Health Risk Assessment

**DOI:** 10.3390/foods15111937

**Published:** 2026-05-30

**Authors:** Nana Sun, Rui He, Yongfu Shi, Ruolin Li, Qi Li, Xiaoying Zhang, Dongmei Huang, Changling Fang, Feng Han, Liangliang Tian

**Affiliations:** 1 Key Laboratory of Oceanic and Polar Fisheries, East China Sea Fisheries Research Institute, Chinese Academy of Fishery Sciences, Ministry of Agriculture and Rural Affairs P. R. China, Shanghai 200090, China; 2 College of Food Science and Technology, Shanghai Ocean University, Shanghai 201306, China; 3 College of Food Science and Technology, Dalian Ocean University, Dalian 116023, China

**Keywords:** cadmium, *Oratosquilla oratoria*, accumulation characteristics, gonads, mechanisms, health risk

## Abstract

The bioaccumulation of cadmium (Cd) in the edible tissues of *Oratosquilla oratoria*, a seasonal delicacy in Shanghai, poses potential health risks to consumers. This study investigated Cd accumulation in the edible tissues (muscle, gonad, hepatopancreas, intestine, heart) of *Oratosquilla oratoria* purchased from Shanghai markets, focusing on cadmium content during gonadal development using inductively coupled plasma mass spectrometry (ICP-MS). Results revealed the hepatopancreas as the primary site of Cd storage, with concentrations in the intestine and hepatopancreas (10.64–58.27 mg/kg) being orders of magnitude higher than those in the gonads and muscle (0.01–0.48 mg/kg). Strikingly, Cd levels in the gonads remained consistently low throughout development and did not correlate with the gonadosomatic index. This indicates a biological barrier that sequesters Cd in the outer gonad membrane of *Oratosquilla oratoria*, thereby protecting germ cells from toxicity. Health risk assessment indicated that consumption of the intestine and hepatopancreas poses a potential carcinogenic risk, whereas the most commonly consumed tissues—muscle and gonads—present a low risk. Our findings clarify the differential bioaccumulation of Cd in *Oratosquilla oratoria*, reveal a potential biological mechanism for gonadal protection, and provide a scientific basis for targeted consumption guidance to mitigate public health risks.

## 1. Introduction

*Oratosquilla oratoria*, also known as “mantis shrimp” or “squilla”, belongs to the phylum Arthropoda, class Crustacea, order Stomatopoda, family Squillidae, and genus *Oratosquilla*. It is widely distributed in the coastal areas of China, Japan, and the Philippines. It is commonly found in burrows on muddy seabeds in shallow coastal waters and is a common organism in the sea areas around coastal cities [1]. In Shanghai, the consumption of *Oratosquilla oratoria* shows clear seasonal characteristics. Every year, the most fertile period of *Oratosquilla oratoria* is from April to May, just before the fishing ban period. Female shrimp contain abundant roe, and male shrimp have plump flesh, making it a seasonal delicacy on the tables of Shanghai citizens. The primary market for *Oratosquilla oratoria* includes Shanghai, a metropolitan area with a population of 26 million. Consumers in Shanghai commonly ingest various edible tissues of the mantis shrimp together, with the hepatopancreas and gonads being particularly favored for their flavor and texture. With increasing consumption of *Oratosquilla oratoria*, the problem of heavy metal cadmium pollution has gradually attracted attention. A number of studies have shown that mantis shrimp have a very strong bioaccumulation capacity for cadmium. For instance, Zhao et al. [2] reported a 100% exceedance rate for cadmium in the edible tissues of samples from Shanghai. Furthermore, the cadmium content in the gonads is approximately ten times higher than that in the muscle tissue [3]. Cadmium inhibits enzyme activity by binding to proteins containing carboxyl and amino groups, damages the kidneys, bone marrow, and digestive system, reduces immunity, and causes osteoporosis (as seen in the Japanese “Itai-Itai disease”) [4]; it also interferes with metal ion metabolism and increases the detoxification burden on organs [5]. In addition, the International Agency for Research on Cancer has classified cadmium as a Group 1 carcinogen [6]. In recent years, the focus of consumers in Shanghai regarding mantis shrimp has gradually shifted from simple freshness and taste to safety and health risks. This shift makes the risk assessment of cadmium in mantis shrimp particularly important.

Most existing research on cadmium (Cd) accumulation in *Oratosquilla oratoria* has been conducted from a food safety perspective. Because of the anatomical difficulty of completely separating the hepatopancreas from the gonads, these studies have typically analyzed the tissues together and reported high overall Cd levels. Consequently, this approach has obscured the specific distribution pattern of Cd between these organs, leaving it unclear—and indeed controversial—whether the gonads themselves are a primary site of accumulation. This question is particularly pertinent given that *Oratosquilla oratoria* is a representative commercial species in Shanghai, where consumer exposure risk is a primary concern. Moreover, although it is known that material exchange and metal accumulation occur during gonadal development, systematic data on Cd content in different tissues across developmental stages are still lacking. Thus, a comprehensive investigation into the Cd accumulation characteristics in various tissues, especially the changes in Cd levels throughout sexual maturation, is critically needed.

Therefore, this study was designed to address these gaps. We meticulously dissected fresh *Oratosquilla oratoria* to completely separate the hepatopancreas, gonads, muscle, and heart, and employed ICP-MS to accurately quantify Cd concentrations in these tissues from specimens collected in Shanghai markets. Our objectives were to: (1) elucidate the differential Cd accumulation characteristics across these tissues, with a particular focus on its dynamic changes during gonadal development; (2) compare Cd accumulation between males and females; and (3) assess the potential carcinogenic and non-carcinogenic health risks associated with consuming different edible parts. The findings will provide a scientific basis for more targeted consumption guidance and offer new insights into the potential mechanisms of Cd interception and detoxification in *Oratosquilla oratoria*.

## 2. Materials and Methods

### 2.1. Instruments and Reagents

Nitric acid (analytical grade), J.T. Baker, Phillipsburg, NJ, USA; Cadmium standard solution (10 μg/mL), Agilent Technologies, Santa Clara, CA, USA; Indium (In) internal standard solution (10 μg/mL), Agilent Technologies, USA.

Agilent-7500 Inductively Coupled Plasma Mass Spectrometer, Agilent Technologies, USA; Ethos 1/A Microwave Digestion System, Milestone, Sorisole, Italy; 16RXII High-Speed Refrigerated Centrifuge, Hitachi CF, Tokyo, Japan; HP-H35SC Electric Heating Plate, Shengda Jieshen Automation Equipment Co., Ltd., Jinan City, China; Milli-Q ultrapure water purification system, Millipore, Burlington, MA, USA. All experimental vessels, including PTFE digestion vessels, were immersed in 35% nitric acid for 48 h, thoroughly rinsed with ultrapure water, air-dried, and then used.The microwave digestion procedure is shown in Table 1.

### 2.2. Sample Collection and Target Tissue Dissection

**Figure 1:**
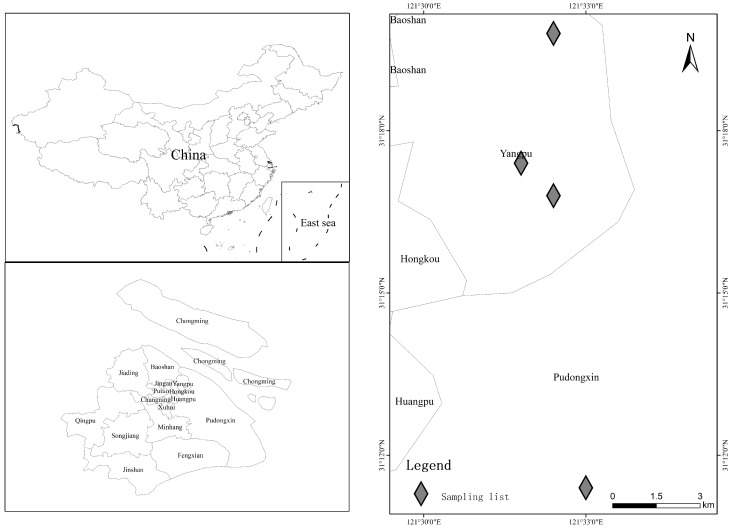
Study area and sampling locations of *Oratosquilla oratoria* from shanghai.

From April to September 2025, this study collected a total of 87 *Oratosquilla oratoria* specimens (including 35 males). All specimens were purchased from supermarkets and aquatic markets in Yangpu District, Shanghai. This is illustrated in Figure 1. No concurrent measurements of Cd concentrations in sediment or water were available, as samples were purchased directly from markets. The collected *Oratosquilla oratoria* weighed between 14.53 g and 49.64 g, with an average weight of 29.99 g. Purchased specimens were maintained under low-temperature aerobic conditions (12~15 °C) and dissected on the same day to ensure they remained alive during dissection.

**Figure 2:**
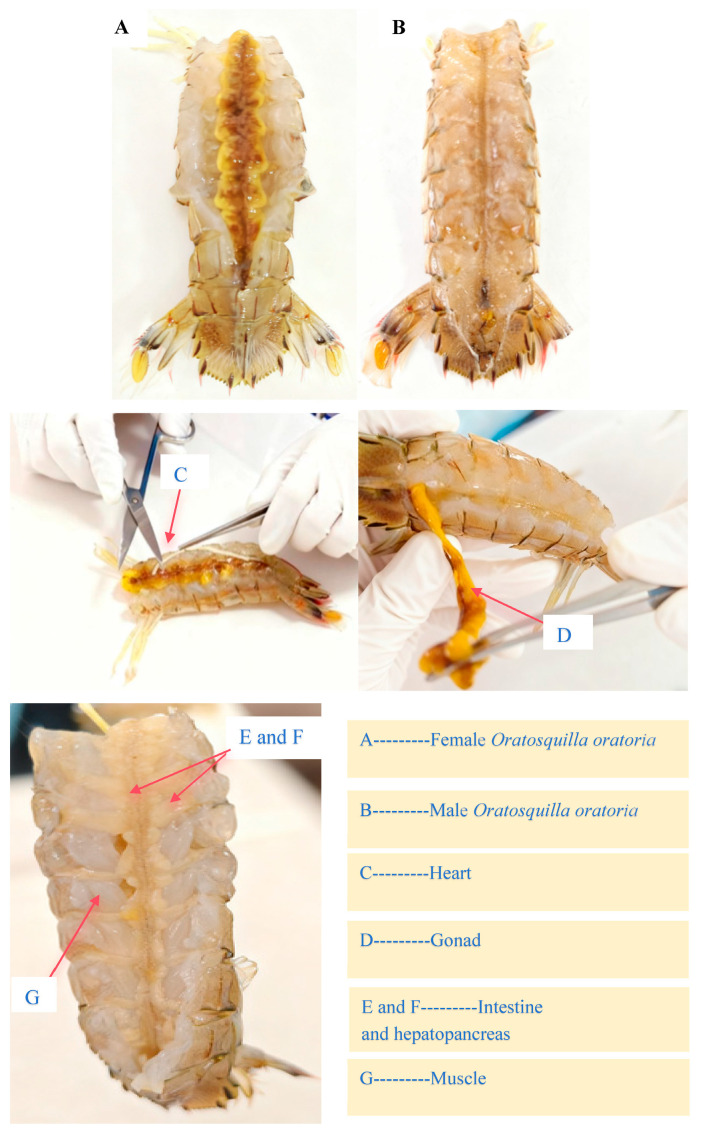
(**A**) Schematic diagram of the internal structure of female *Oratosquilla oratoria*. (**B**) Schematic diagram of the internal structure of male *Oratosquilla oratoria*.

During dissection, clean impurities from the crab’s surface using ultrapure water, then blot dry with paper towels. Weigh the total mass of the *Oratosquilla oratoria*. Use scissors to cut open its dorsal side, remove the carapace, and peel away the muscles. A long, white, tubular heart will be visible along the dorsal midline. Carefully extract the heart using tweezers and scissors. Considering that the tissues of the *Oratosquilla oratoria* begin to dissolve and decay once the heart is removed, it is essential to promptly separate the gonads, intestines, and hepatopancreas. Use tweezers to remove the gonads. After removal, the spider-web-like hepatopancreas becomes visible. This organ encloses the intestine internally and extends laterally from the left and right sides at the segmental junctions toward the body margins [7,8]. Divide the dissected *Oratosquilla oratoria* tissues into four groups: (1) Muscle; (2) Gonads; (3) Intestines and Hepatopancreas; (4) Heart. To prevent cross-contamination between tissues, clean dissection tools after removing each tissue before proceeding to the next. Following dissection, edible tissues from male *Oratosquilla oratoria* were blended and homogenized, with female specimens undergoing identical processing. Homogenized samples were stored at −20 °C for subsequent analysis. All cadmium measurements are reported on a wet weight basis. Anatomical images of male and female *Oratosquilla oratoria* are shown in Figure 2A,B.

According to relevant research reports, the growth and development of the female *Oratosquilla oratoria* ovary can be divided into six stages (Table 2), while the development of the male testis can be divided into four stages (Table 3).

The male *Oratosquilla oratoria* collected in this study were all in the sexually mature stage (stage IV), while the female *Oratosquilla oratoria* samples were collected from stages VI to VIII. Due to the small number of female *Oratosquilla oratoria* in the early stage, and the connection between the gonads and the hepatopancreas in this stage is easily damaged and difficult to separate, the cadmium content of the samples in the early stage was not tested. All tissue samples were homogenized, weighed, and stored at −20 °C. Cadmium content analysis was based on wet weight data.

### 2.3. Sample Preparation and Analysis

Determination of cadmium content in *Oratosquilla oratoria* was conducted according to Method 1: Inductively Coupled Plasma Mass Spectrometry (ICP-MS) in GB 5009.268-2016 “National Food Safety Standard: Determination of Multiple Elements in Food” [10]. According to the standard, samples were weighed and digested. Cadmium quantification employed seven levels of standard working solutions (0, 0.1, 0.2, 0.5, 1.0, 2.0, and 5.0 ng/mL) as internal standards. The R^2^ of the calibration curve is 0.9998. The method has a limit of detection (LOD) of 0.02 mg/kg and a limit of quantification (LOQ) of 0.05 mg/kg.

### 2.4. Gonadal Index

Before dissection, the surface of the *Oratosquilla oratoria* was dehydrated by the standard water absorption method (Whatman No. 1 filter paper). The weight of the whole *Oratosquilla oratoria* was first weighed, and then the gonadal tissue was weighed and stored in a −20 °C refrigerator for analysis. The gonadosomatic index (GSI) was calculated according to the following formula [11]:

GSI (%) = Gonad wet weight/Body wet weight × 100

### 2.5. QA/QC Control

A razor clam (*Sinonovacula constricta*) quality control sample (national certified reference material, provided by the Second Institute of Oceanography, State Oceanic Administration) was used to ensure quality control throughout the analytical process and results, including precision tests, repeatability tests, and accuracy tests. The quality control analysis was performed in triplicate to confirm that the analytical method employed is accurate and reliable for the determination of total cadmium in the tested samples. Data are expressed as the mean ± standard deviation.

The total cadmium content in a certified reference material of *Sinonovacula constricta* (razor clam) was determined by ICP-MS. The measured value of cadmium was 0.37 ± 0.01 (n = 3), which falls within the certified reference range of 0.38 ± 0.02 mg/kg, with a relative standard deviation (RSD) of less than 5%. This indicates good accuracy of the test results. Therefore, the method employed in this study is confirmed to be accurate and reliable.

### 2.6. Method for Assessment of Cadmium Pollution

The single-factor pollution index (Pi) for heavy metals in aquatic products is used to evaluate the pollution level of a single heavy metal element in aquatic products. The calculation formula is as follows [12]:

$$Pi=\frac{Ci}{Si}$$

Ci denotes the mean value of heavy metal element i in aquatic products, measured in mg/kg; Si denotes the maximum permissible limit for heavy metal i in aquatic products, measured in mg/kg. Evaluation standards refer to the “National Food Safety Standard: Maximum Contaminant Levels in Foods” (GB 2762-2022) [13].

Evaluation Criteria: Pi < 0.2 indicates normal background levels; 0.2 ≤ Pi < 0.6 indicates mild pollution levels; 0.6 ≤ Pi < 1.0 indicates moderate pollution levels; Pi ≥ 1.0 indicates severe pollution levels.

### 2.7. Human Dietary Exposure Risk Assessment Method

Estimated daily intake (EDI) represents the amount of heavy metals ingested by humans from *Oratosquilla oratoria* daily [14], expressed as micrograms per kilogram of body weight per day (μg/kg bw/d):

$$EDI=\frac{EF\times ED\times I\times C}{BW\times AT}$$

In the formula: EF represents exposure frequency (365 days/year); ED (Exposure Duration) denotes continuous exposure time (70 years, equivalent to average human lifespan); I (Intake) represents the average daily intake in g/d. Since the Sixth China National Dietary Intake Survey did not include lifetime average consumption data for *Oratosquilla oratoria* in Shanghai, and given Suzhou’s geographical proximity and similar dietary habits to Shanghai, Suzhou’s annual average consumption of *Oratosquilla oratoria* is 3.03 kg [15]. Therefore, the average daily intake of *Oratosquilla oratoria* in Shanghai is 8.3 g/d; C (Concentration) represents the concentration of heavy metals in seafood (mg/kg, bw); BW (Body Weight) denotes the average body weight of an adult human (assumed to be 60 kg) [16]. AT (Average Time) represents the average exposure time for non-carcinogenic substances, calculated in this study as 70 years (365 days/year × 70 years).

Target Hazard Quotients (THQ) is a risk assessment method published by the U.S. Environmental Protection Agency for evaluating human exposure to exogenous pollutants through dietary intake. Target Hazard Quotients serve as a method for assessing dietary risks to humans and are widely used in health risk assessments for heavy metal pollutants [17]. This study employs the point assessment method for high-end cadmium boundary evaluation and utilizes the target hazard quotient method to assess the health risks of cadmium exposure in *Oratosquilla oratoria*. When the total hazard quotient is less than 1, it indicates no significant dietary risk for the exposed population; conversely, a risk exists when the quotient exceeds 1 [18]. The higher the THQ value, the greater the risk level.

$$THQ=\frac{EDI}{RfDo}$$

In the formula: EDI denotes the estimated daily intake of heavy metals in humans, expressed in mg/kg day; RfDo represents the reference dose for daily exposure, at which humans can be continuously exposed throughout their lifetime without a substantial carcinogenic risk [19]. The U.S. Environmental Protection Agency (US EPA) recommends an RfDo of 0.001 mg/kg day [20].

Target cancer risk (TCR) is an indicator used in environmental health and food safety to quantify the probability of excess cancer incidence resulting from lifetime exposure to potential chemical carcinogens [21]. A TCR greater than 10^−4^ indicates a higher risk of cancer; a TCR between 10^−4^ and 10^−6^ indicates a risk of cancer; if the TCR is less than 10^−6^, the cancer risk can be considered negligible. The TCR calculation formula is as follows [15]:

$$TCR=EDI\times CSFo\times 10^{-3}$$

In the formula: EDI denotes the estimated daily intake of heavy metals via oral exposure in humans, expressed in mg/kg day; CSFo represents the carcinogenic slope factor for oral intake, with the value for cadmium being 0.38 (mg/kg day)^−1^ [22].

### 2.8. Statistical Analysis

Data analysis was performed using Microsoft Excel 2016 and SPSS (version 24.0) software. Results are expressed as means ± standard deviation (SD). Plots were generated using Origin 2024 software.

## 3. Results and Discussion

### 3.1. Sample Determination Results and Analysis

#### 3.1.1. Cadmium Content Characteristics in Different Tissues

Table 4 shows the cadmium content in different edible tissues of the *Oratosquilla oratoria*. Cadmium levels in the intestines and hepatopancreas were significantly higher than in other tissues, with concentrations decreasing in the following order: intestines and hepatopancreas > heart > muscle > gonads.

**Figure 3:**
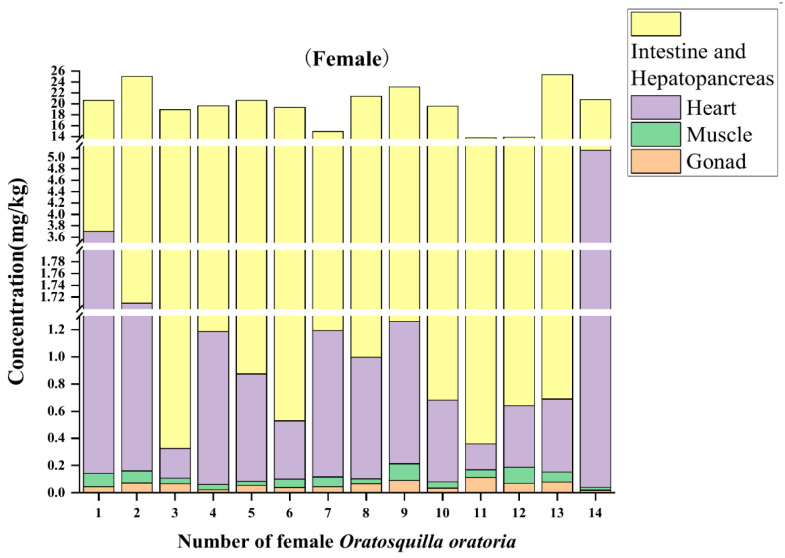
Cadmium accumulation in different edible tissues of female *Oratosquilla oratoria*.

**Figure 4:**
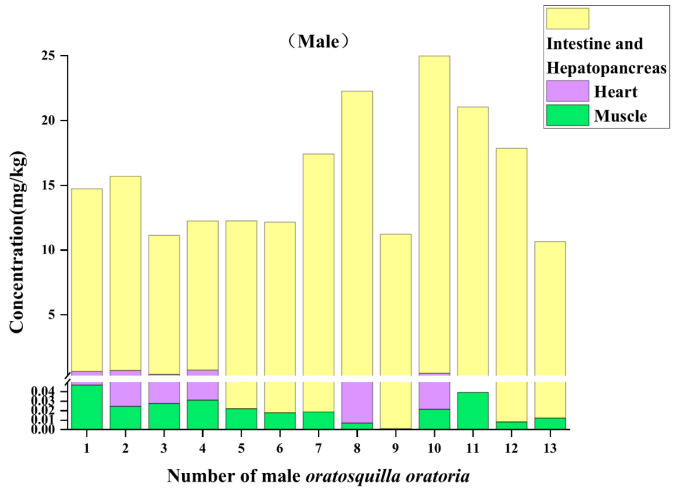
Cadmium accumulation in different edible tissues of male *Oratosquilla oratoria*.

*Oratosquilla oratoria* are a highly popular edible marine seafood. Xu Xin et al. [23] found that all *Oratosquilla oratoria* encountered in fishery surveys originated from wild populations, with no records of artificial cultivation. According to data from the 2024 China Fishery Statistical Yearbook, the national catch of *Oratosquilla oratoria* in 2023 reached 227,265 tons, representing a 2.37% increase compared to 2022. However, the food safety issues concerning heavy metals in the edible tissues of *Oratosquilla oratoria* have also drawn widespread attention. Due to their benthic lifestyle, crustacean aquatic organisms like *Oratosquilla oratoria* are more prone to specifically accumulating toxic substances such as heavy metal cadmium. These substances can then be transmitted through the food chain and bioaccumulate in the human body, posing greater safety risks when consumed [24]. Their breeding season spans from April to August, with peak sexual maturity occurring in two periods: April to May and July to August. The *Oratosquilla oratoria* products during the sexual maturity period are more favored by consumers, hence the cadmium content in the gonadal tissues of mantis shrimp has attracted significant research attention. Research findings indicate that cadmium concentrations (wet weight) are higher in the intestine and hepatopancreas, while lower concentrations are observed in the gonads and muscle tissue. Consequently, significant variations in cadmium content exist across different edible tissues. The hepatopancreas serves as the crustacean’s primary digestive gland, performing both hepatic and pancreatic functions. The liver contains metallothionein, metallothionein-like proteins and nitrogen-containing heterocyclic small molecule compounds. The sulfhydryl group of metallothionein and the ligands of small molecule compounds have high affinity and complexing ability for heavy metals such as cadmium, so the cadmium accumulation is high [25]. The digestive tract, where *Oratosquilla oratoria* digest food, temporarily store nutrients, and excrete waste, shows higher cadmium content, indicating that cadmium primarily enters the mantis shrimp’s body through dietary intake. Consequently, its accumulation concentration is significantly higher than that of other tissues [26]. However, there are few relevant exposure experiments that point to the cadmium accumulation pathways in this species. This result is consistent with previous research by Han Dianfeng [24] indicating that the digestive tract is the primary tissue for cadmium accumulation in *Oratosquilla oratoria*, with cadmium accumulation concentrations and enrichment rates significantly higher than those in muscle and gonads. Additionally, this study is the first to determine cadmium levels in the gonads and other tissues of *Oratosquilla oratoria*, revealing extremely low cadmium concentrations in the gonads. Detailed data are shown in Figure 3 and Figure 4.

**Figure 5:**
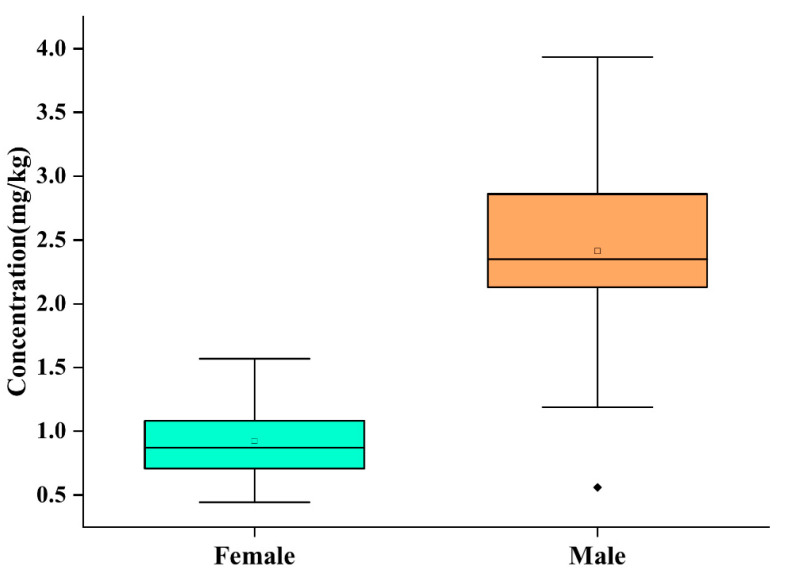
Cadmium concentration in mixed edible tissues of female/male *Oratosquilla oratoria*.

Figure 5 compares the cadmium (Cd) concentrations between female and male *Oratosquilla oratoria*. The results demonstrate that males exhibit significantly higher Cd burdens than females, indicating a stronger Cd-bioaccumulation capacity in the male cohort. This is consistent with the findings reported by Wu et al. Cadmium is primarily detoxified in vivo via complexation with metallothionein-like proteins (MTLP); the observed higher Cd levels in males may therefore reflect a more robust MTLP-binding capacity, facilitating greater internal sequestration of the metal. At present, studies addressing sex-specific Cd accumulation in *Oratosquilla oratoria* remain scarce, and the underlying biological mechanisms await further elucidation.

#### 3.1.2. Variation Features of Cadmium Content in *Oratosquilla oratoria* Across Developmental Stages

The gonadosomatic index (GSI) is a critical indicator for revealing the reproductive status of a species and is widely used to determine the reproductive period in aquatic animals [9]. Sexual maturity is an important stage in the development of fishery resources, and it is an important turning point for fishery resources to develop from larvae to adults and begin to participate in reproductive activities [27]. At present, there are few studies on the relationship between the first sexual maturity and body size and age in crustaceans, and only a few studies have confirmed that the gonadal index in red-eyed shrimp is negatively correlated with body weight [28].

**Figure 6:**
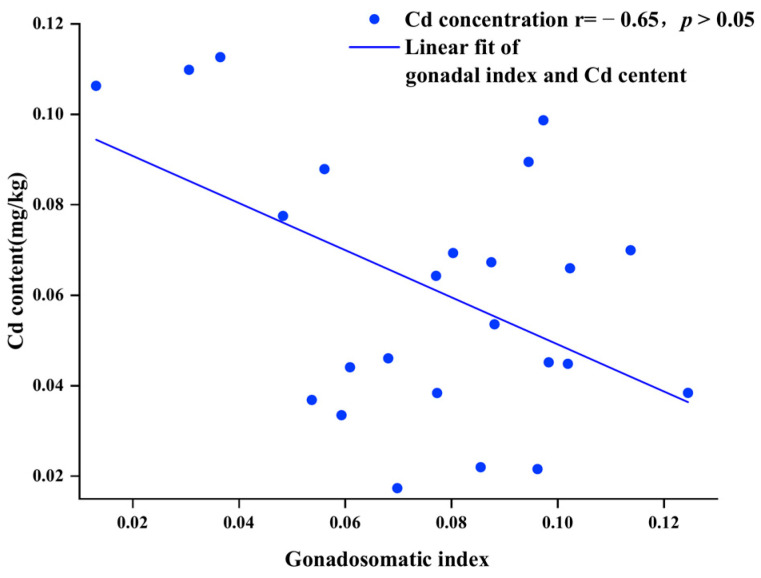
Linear fit of Gonadosomatic Index and Cadmium Concentration.

The gonadal index of the *Oratosquilla oratoria* serves as a crucial biological indicator, primarily used to assess the degree of gonadal development in individual specimens, determine their reproductive season, and investigate their reproductive biology. In this study, the gonadal index of female Chinese *Oratosquilla oratoria* wet specimens ranged from 1.31% to 12.45%, with an average value of 7.25%. As shown in Figure 6, the gonadal index of *Oratosquilla oratoria* exhibits an overall negative correlation with cadmium content.

**Figure 7:**
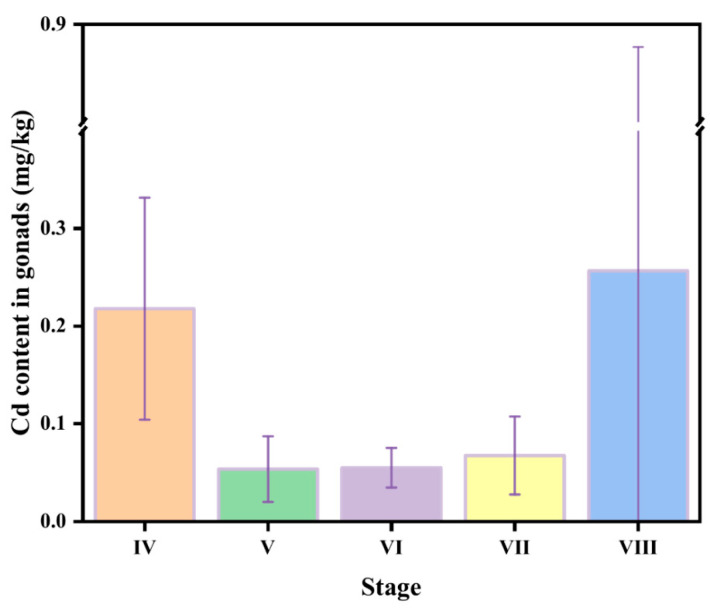
Cadmium in the ovary at different stages of ovarian development.

**Figure 8:**
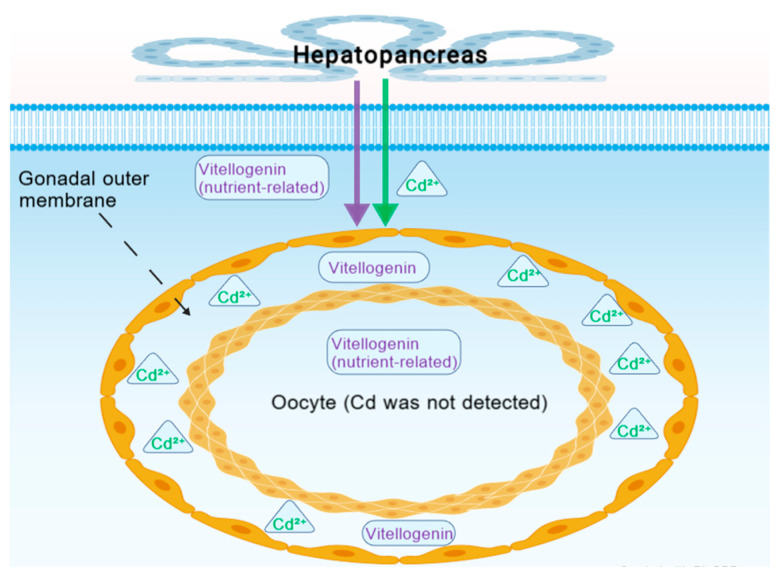
“Nutrient–toxicity shunt” mechanism: the vitellogenin from the hepatopancreas was efficiently taken up by the ovary, while the cadmium–MT complex was secondarily captured and fixed in the outer membrane, thereby blocking the toxic pathway to germ cells and ensuring normal embryonic development.

Combining the changes in ovarian growth and cadmium contentin in female mantis shrimp, we observed the pattern of cadmium accumulation during gonad development (Figure 7). Figure 8 shows the physiological structure of the female mantis shrimp gonad at different developmental stages. The ovary is the main reproductive organ of the female shrimp, and its development directly affects the reproduction of the offspring [29]. The ovary of the mantis shrimp is mainly composed of the ovary, oviduct and seminal receptacle. The outer membrane is a special stratified epithelium that produces oocytes and follicular cells in a radial manner [30]. Ovarian development includes the differentiation of oocytes and the accumulation of yolk, which leads to corresponding changes in the size and appearance of the gonads.

The cadmium content in the gonads of female shrimp is not constant, nor does it simply increase with the accumulation time. In the early and late stages of ovarian development, the cadmium level in the gonads is higher than that in other stages, especially in stages IV and VIII, which is significantly higher than that in other stages. This phenomenon may be related to the characteristics of ovarian development and the change in gonadal somatic index (GSI) of *Oratosquilla oratoria*. During the rapid development of gonads (stages V–VII), oocytes proliferate in large quantities and accumulate yolk substances, resulting in the deepening of the yellow appearance of the ovary, the increase in the overall volume, and the increase in GSI. The cadmium concentration in the gonads is extremely low at this stage.

During Phase VII, with the release of a large number of mature eggs, the ovarian volume decreases. However, at this stage, the cadmium concentration in the gonads soars to the highest point throughout the reproductive cycle, far exceeding that in any other period. The rise in ovarian cadmium levels during the ovulation process may suggest that the external structures of the ovary have retained most of the cadmium within the overall ovarian tissue. This is indirectly supported by the extremely low cadmium content found in the oocyte.

Cadmium exhibits significant reproductive toxicity to organisms [24]. However, this study indicates that as the gonads continue to develop, the cadmium content within them does not show a significant increasing trend, suggesting that the gonads of *Oratosquilla oratoria* do not possess the ability to accumulate cadmium. The hepatopancreas is the primary site for the synthesis of structural proteins such as vitellogenin. These proteins are subsequently transported via the hemolymph to the ovaries, where they are utilized for oocyte development and yolk deposition [31]. The research findings reveal that the cadmium content is relatively high in the hepatopancreas and digestive tract, indicating that cadmium does not enter the ovarian part of the gonads along with nutrients like proteins. This result suggests that there may exist a certain blocking mechanism in the gonads of *Oratosquilla oratoria* that retains cadmium in the outer membrane of the ovaries, preventing its entry into the reproductive cells and thus not affecting the production and development of the shrimp’s offspring.

#### 3.1.3. A Hypothesis on Cadmium Interception in *Oratosquilla oratoria*

Previous research has often attributed cadmium accumulation in crustaceans to environmental factors, overlooking their physiological regulation [32]. In the *Oratosquilla oratoria*, the gonad has been reported to accumulate Cd at concentrations roughly ten times higher than those in muscle, and the majority of this Cd is bound in organic forms, with the highly toxic inorganic ion fraction typically below 20% [33]. During gonadal maturation, the hepatopancreas synthesizes and secretes large quantities of vitellogenin (VTG) and other nutritional proteins, which are transported through the hemolymph to the ovary to support oocyte development. In decapod crustaceans, the specific uptake of VTG by oocytes occurs via vitellogenin receptor (VgR)-mediated endocytosis; this receptor system has recently been reconceptualized as a selective molecular filter capable of buffering oocytes from toxic challenges during vitellogenesis [34].

To mechanistically explain this phenomenon, we propose a hypothetical “nutrient–toxicity shunt” model. Specifically, we hypothesize that during the massive hemolymph-borne transport of hepatopancreas-derived substances to the ovary, VTG is efficiently taken up into oocytes via VgR-mediated endocytosis, whereas the Cd–MT complex is largely excluded from this pathway. We further speculate that Cd–MT is secondarily captured and sequestered by the follicular cells, possibly through alternative receptors such as scavenger receptors or megalin/cubilin, thereby establishing a safeguard that physically separates Cd from the developing oocytes. If operative, this putative barrier would theoretically protect developing embryos from Cd toxicity during oogenesis and subsequent embryogenesis. Notably, Cd has been shown to disrupt VTG synthesis in the hepatopancreas and thereafter inhibit ovarian development in the red swamp crayfish *Procambarus clarkii*, indicating that the VTG transport axis is indeed vulnerable to Cd interference and underscoring the biological plausibility of an ovarian-level countermeasure [35].

Cd enters the hepatopancreas via Ca^2+^ channels/DMT1 and induces MT binding. While VTG and other nutritional proteins synthesized by the hepatopancreas are massively transported through the hemolymph to meet the demands of gonadal development, the Cd–MT complex does not accompany VTG, or even if it reaches the ovary, it is recaptured by the outer follicular cell layer. Experimental observations show that Cd remains almost completely in the ovarian outer membrane (follicular cell layer), with extremely low concentrations inside the oocytes. This supports the existence of a “nutrient–toxicity shunt” mechanism: VTG from the hepatopancreas is efficiently taken up by the ovary, whereas the Cd–MT complex is secondarily captured and immobilized in the outer membrane, thereby blocking the toxic route to germ cells and ensuring normal embryonic development.

It must be emphasized, however, that the “nutrient–toxicity shunt” model is at present speculative and awaits direct experimental validation. The evidence we present and the supporting literature discussed above provide only indirect and correlative clues; no molecular, histological, or biochemical verification—such as immunohistochemical co-localization of MT with the follicular cell layer, isotope or fluorescent tracer studies tracking the differential trafficking of VTG and Cd in vivo, or identification and functional characterization of the follicular receptors putatively responsible for Cd–MT capture—has yet been obtained.

Rather than claiming to have elucidated a verified mechanism, this study offers a clearly defined and experimentally testable hypothesis. It shifts the research focus from traditional food safety risk assessment toward a more mechanistic exploration of how crustaceans may actively partition toxic metals away from reproductive tissues. We argue that the ovary of *Oratosquilla oratoria* might not simply be a site of Cd accumulation but could harbor a regulated filter system that discriminates between essential nutritional proteins and potentially harmful metal complexes. This conceptual framework provides a concrete, experimentally addressable direction for future investigations, which should prioritize the direct localization of Cd–MT complexes, in vivo tracing of VTG and Cd trafficking, and receptor identification in the follicular layer. The validity of the “nutrient–toxicity shunt” hypothesis must now be tested through these targeted approaches. This study thus provides reference data and research directions for future studies on cadmium accumulation mechanisms in aquatic organisms.

### 3.2. Cadmium Pollution Assessment Results

Table 3 shows the cadmium contamination levels in different tissues of *Oratosquilla oratoria*. Current research findings on cadmium contamination in various tissues of *Oratosquilla oratoria* do not fully reflect the distribution patterns of cadmium in the surrounding environment. The midgut and hepatopancreas exhibit the highest cadmium accumulation in edible parts of *Oratosquilla oratoria*. The hepatopancreas serves as the primary metabolic gland in crustaceans, involved in nutrient conversion, synthesis, decomposition, and excretion. The gut functions as the main site for food digestion, and the complexes formed there are difficult to degrade. These factors contribute to cadmium biomagnification within organisms, as both organs are interconnected with food chains and food webs.

In the newly revised National Food Safety Standard: Maximum Contaminant Limits in Foods (GB 2762-2022) [13], separate provisions have been established for cadmium limits in two crustacean products—*Oratosquilla oratoria* and sea crabs—raising the threshold to 3 mg·kg^−1^. The results are presented in Table 5. While muscle, gonads, and heart tissues of *Oratosquilla oratoria* did not exceed the limit, their intestines and hepatopancreas exceeded the relevant standard. Notably, evaluation results indicate that cadmium contamination levels in the intestines and hepatopancreas of *Oratosquilla oratoria* reached heavily polluted levels. Therefore, it is necessary to conduct further dietary exposure risk assessments for different tissues through human health risk evaluations.

### 3.3. Human Health Risk Assessment

**Figure 9:**
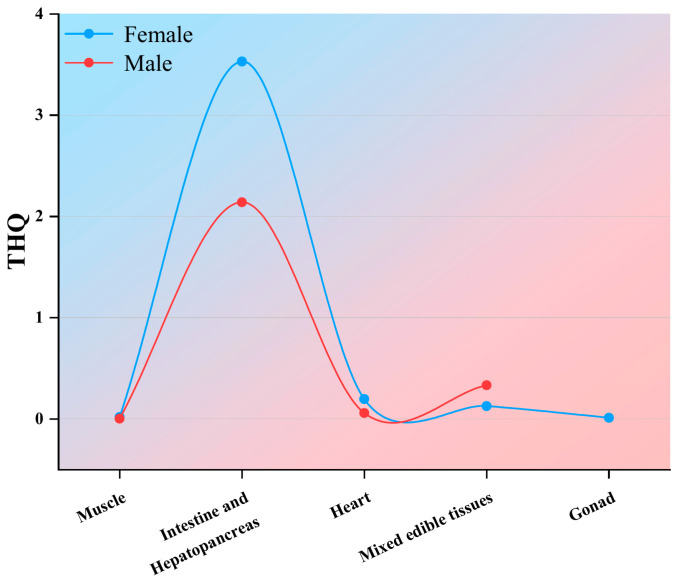
Hazard quotients of Cd via consumption of the different edible tissues of *Oratosquilla oratoria*.

**Figure 10:**
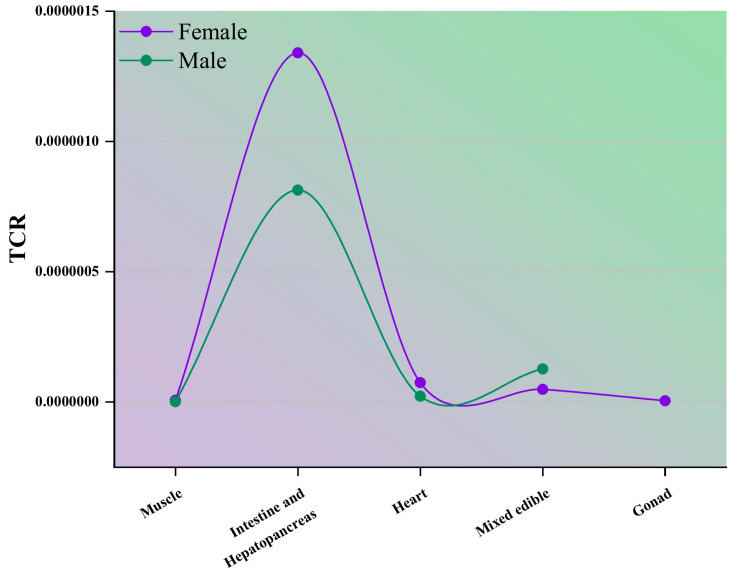
Target cancer risk of Cd via consumption of the different edible tissues of *Oratosquilla oratoria*.

The dietary exposure risk assessment of cadmium holds significant importance for evaluating the health risks associated with local residents’ consumption of Shanghai *Oratosquilla oratoria*. Two risk assessment methods—target hazard quotient (THQ) and target cancer risk (TCR)—were employed to quantify the non-carcinogenic and carcinogenic risks posed by Shanghai *Oratosquilla oratoria*. This is illustrated in Figure 9 and Figure 10.

A study on cadmium levels in different parts of *Oratosquilla oratoria* collected from Shanghai markets and an associated dietary risk assessment. Results (Table 4) indicate that dietary exposure risk to cadmium is highest in the intestine and hepatopancreas, while the lowest risk is found in the gonads and muscle tissue. Notably, TCR analysis of the intestine and hepatopancreas revealed potential carcinogenic risks that warrant attention. The dietary exposure risk index (THQ) indicates that consuming the muscle tissue carries significantly lower dietary exposure risk than consuming the intestine or hepatopancreas. The assessment of cadmium contamination levels in different tissues and human consumption safety indicates that consuming the intestine and hepatopancreas carries higher risks than consuming the gonads and muscle tissue. This finding is consistent with the research conducted by Li Jie [36] and Lu Furong [37], which demonstrated that the Target Hazard Quotient (THQ) decreases following the removal of the digestive gland, suggesting a higher risk of cadmium exposure associated with the consumption of mantis shrimp when the digestive gland is retained. Furthermore, consuming male *Oratosquilla oratoria* carries slightly higher risks than consuming female *Oratosquilla oratoria*, with similar findings observed in target cancer risk results. As one of China’s most common economically important shrimp species, we place particular emphasis on the potential bioaccumulation of cadmium in different edible tissues. Furthermore, the exposure risks associated with consuming different edible tissues warrant additional attention. Detailed data are shown in Table 6.

Cadmium, as a toxic heavy metal, accumulates in marine organisms and can induce various toxic effects such as oxidative stress, neurotoxicity, and growth inhibition, posing potential risks to aquatic ecosystems [38]. Cadmium entering coastal environments through rivers and atmospheric deposition as a result of industrial and agricultural activities readily accumulates in aquatic organisms, particularly in benthic organisms. Through the amplification effect of the food chain, it ultimately poses a threat to the health of humans as the terminal consumers [38]. Therefore, seafood consumption, particularly among coastal populations where seafood constitutes a significant portion of the diet, represents a major pathway for cadmium exposure. The migration and transformation of cadmium in marine environments are significantly influenced by its chemical form and sediment characteristics [38], while its bioaccumulation efficiency is closely related to species-specific physiological and ecological traits, exposure pathways, and their position within the food web [39]. This complex biogeochemical process makes seafood a key conduit for cadmium entering the human body from the environment, increasing the likelihood of chronic health damage—such as nephrotoxicity—from long-term dietary exposure [40]. Therefore, conducting cadmium contamination studies on specific economic species is crucial for accurately assessing health risks.

In estuarine and coastal areas significantly impacted by human activities, cadmium levels in sediments often increase, leading to benthic organisms inhabiting these regions becoming highly enriched cadmium populations. Table 7 presents cadmium (Cd) concentrations in sediments from various regions of China, including bays, estuaries, and rivers. Using the *Oratosquilla oratoria* commonly found in Shanghai markets as a study subject, analyzing cadmium concentrations in its various tissues not only indicates the cadmium pollution background in the Yangtze River Delta estuary region but also directly assesses the health risks local residents face through dietary intake. Research has consistently confirmed that the visceral organs of crustaceans (such as hepatopancreas and intestinal glands) serve as primary accumulation sites for heavy metals. Consumers can effectively reduce cadmium exposure by selectively consuming muscle tissue. This dietary recommendation is supported by food safety risk assessments in multiple countries [41]. Furthermore, this study reveals extremely low cadmium levels in the neutral gland tissue of *Oratosquilla oratoria*, confirming that consumers can safely consume this part. This study provides scientific evidence for formulating targeted consumption recommendations and regional food safety regulations by analyzing cadmium distribution differences across edible parts of *Oratosquilla oratoria*.

## 4. Conclusions

This study investigated the differences in cadmium accumulation in the edible tissues of *Oratosquilla oratoria* from Shanghai markets. The results indicate that *Oratosquilla oratoria* exhibits cadmium accumulation capacity, although accumulation varies among body parts. Cadmium levels in the gonads are extremely low, while the intestine and hepatopancreas serve as the primary cadmium storage sites. These results clearly show for the first time that the gonads of *Oratosquilla oratoria* do not accumulate cadmium, nor is the metal passed on to future generations. Because the reproductive glands contain almost no cadmium, we speculate that a “nutrient–toxicity shunt” mechanism exists in the body to ensure reproductive safety. In addition, many unresolved questions remain regarding the cadmium accumulation mechanism in *Oratosquilla oratoria*. Future research directions may include morphological analysis of cadmium, exploration of cell signaling pathways, and studies on the expression of related proteins. Health risk assessment revealed that for the general consumer population, the target hazard quotient (THQ) for cadmium exposure from consuming only muscle and gonadal tissues falls within acceptable limits, indicating low non-carcinogenic health risks. However, consumption practices that include the intestine and hepatopancreas pose clear potential health risks. These risks are particularly pronounced for sensitive groups such as high-frequency consumers, children, and pregnant women. This study reveals the bioaccumulation of cadmium in *Oratosquilla oratoria*, providing guidance for Shanghai residents consuming this species. It should be noted that systematic data on the lifetime average consumption of *Oratosquilla oratoria* among Shanghai residents are currently unavailable. In contrast, Xu and Li [54] reported that residents in the Yangtze River Delta share common influencing factors for aquatic product consumption (e.g., product preference and purchasing habits), resulting in highly consistent consumption behavior within this region. Given that Suzhou and Shanghai are both located in the Yangtze River Delta, are geographically adjacent, and belong to the same cultural and economic zone, intraregional extrapolation of consumption data from Suzhou to Shanghai is justifiable.

According to the latest revision of national food safety standards, the cadmium limit has been raised from 0.5 mg/kg to 3.0 mg/kg. While this increase has slightly reduced the rate of non-compliance, it does not guarantee food safety. The provisional tolerable monthly intake (PTMI) for cadmium set by the Joint FAO/WHO Expert Committee on Food Additives (JECFA) remains unchanged at 0.025 mg/kg body weight. As cadmium is a heavy metal prone to bioaccumulation in the body, long-term excessive intake poses health risks, particularly affecting organs such as the kidneys and bones. Therefore, strict control of cadmium intake is necessary, along with a reduction in the consumption of highly contaminated parts of *Oratosquilla oratoria* (especially the intestine and hepatopancreas).

### Limitations and Future Directions

The primary limitation of this study lies in the fact that the proposed “nutrient–toxicity shunt” hypothesis, although logically inferred from the observed differential distribution of cadmium across ovarian compartments and from established models of vitellogenin uptake and oogenesis, currently lacks direct molecular, histological, and biochemical validation. Specifically, critical experimental evidence—such as immunohistochemical co-localization of metallothionein within the follicular cell layer, in vivo tracer studies tracking the differential trafficking routes of vitellogenin and cadmium, and functional characterization of the putative follicular receptors responsible for the secondary capture of Cd–MT complexes—has not yet been obtained. The hypothesis must therefore be regarded as a speculative but clearly formulated and testable working model. A secondary limitation concerns the extrapolation of our findings to human health risk assessment. The present study is based on a limited number of samples collected within a confined spatial and temporal window and toxicokinetic models of cadmium bioavailability in humans. Expanding future sampling to broader spatiotemporal scales, systematically monitoring pollution dynamics, and integrating bioavailability assessments would help translate the observed internal partitioning of cadmium into more precise and population-specific exposure estimates.

The current conclusion remains valid for the scope of method establishment.

Accordingly, future research should pursue two parallel priorities: first, targeted molecular and imaging studies designed to directly test the nutrient–toxicity shunt hypothesis, and second, large-scale surveys integrated with advanced bioavailability modeling to refine the risk assessment framework for cadmium in edible crustaceans. Notwithstanding its current limitations, the present study offers a concrete conceptual model and a set of clearly testable predictions that can productively guide both lines of investigation.

## Figures and Tables

**Table 1:** Procedure of microwave digestion.

Procedure	Rising Time/min	Holding Time/min	Temperature/°C	Power/W
Heating	15	/	200	1200
Digestion	/	30	200	1200

**Table 2:** Gonadal developmental stage classification criteria in female *Oratosquilla oratoria* [9].

Stage	Classification Characteristics
I	The ovaries are thin and thread-like; there are a large number of oocytes in the left and right ovaries
II	The ovary is enlarged and band-shaped, with a large number of primary oocytes and a small number of oogonia
III	A small amount of yolk appears, and the ovary is light yellow
IV	The ovary volume increases, and the oocytes are arranged tightly
V	The two sides of the ovary are concave and convex in a wave shape, and the S-shaped proliferation zone begins to appear
VI	The ovary is full, the ovary wall becomes thin, and the early mature oocytes occupy the entire ovarian tissue
VII	The ovary is extremely enlarged, and a yellow triangle appears in the middle of the tail section. The oocyte particles can be seen with the naked eye
VIII	After ovulation, the entire ovary begins to shrink, and the oocytes are sparsely distributed

**Table 3:** Gonadal developmental stage classification criteria in male *Oratosquilla oratoria* [9].

Stage	Classification Characteristics
I	The testis is not developed and only contains spermatogonia
II	The spermatogonia area is wrapped in the spermatocyte area
III	From the inside to the outside, there are sperm, spermatocytes, and spermatogonia. There are fewer sperm
IV	There are a large number of sperm in the testis, and there are also a small number of spermatocytes

**Table 4:** Cadmium accumulation in different edible tissues of *Oratosquilla oratoria* (mg/kg, wet weight).

Gender	Tissue	Min	Max	Mean ± SD
Female	Intestine and Hepatopancreas	10.84	58.27	25.522 ± 11.978
	Gonad	ND	0.41	0.083 ± 0.778
	Muscle	0.02	0.48	0.129 ± 0.117
	Heart	0.09	5.08	1.414 ± 1.323
	Mixed edible tissues	0.44	1.569	0.925 ± 0.386
Male	Intestine and Hepatopancreas	10.64	24.49	15.462 ± 4.793
	Muscle	ND	0.05	0.021 ± 0.030
	Heart	ND	0.70	0.422 ± 0.264
	Mixed edible tissues	0.56	3.93	2.414 ± 0.893

ND: no detected (values below the LOD of 0.02 mg/kg). SD: standard deviation (mg/kg, wet weight).

**Table 5:** Evaluation results of cadmium pollution in edible tissues of *Oratosquilla oratoria*.

Gender	Tissues	S_i_ (mg/kg)	P_i_	Pollution Level
Female	Intestine and hepatopancreas	3	8.507	Heavy pollution
	Gonad		0.028	Normal
	Muscle		0.043	Normal
	Heart		0.471	Light pollution
	Mixed edible tissues		0.308	Light pollution
Male	Intestine and hepatopancreas		5.154	Heavy pollution
	Gonad		0.007	Normal
	Heart		0.141	Normal
	Mixed edible tissues		0.805	Moderate pollution

**Table 6:** Target hazard quotient (THQ) values of cadmium from different edible tissues of *Oratosquilla oratoria*.

Gender	Tissues	Estimated Daily Intake (EDI, mg/kg·day)	Oral Reference Dose (RfDo, mg/kg bw/day)	THQ	Oral Ingestion of Carcinogenic Slope Factor (CSFo, mg/kg/day)	TCR
Female	Intestine and Hepatopancreas	3.53 × 10^−3^	1.00 × 10^−3^	3.53 × 10^0^	3.80 × 10^−1^	1.34 × 10^−6^
	Gonad	1.15 × 10^−5^		1.15 × 10^−2^		4.37 × 10^−9^
	Muscle	1.78 × 10^−5^		1.78 × 10^−2^		6.76 × 10^−9^
	Heart	1.96 × 10^−4^		1.96 × 10^−1^		7.45 × 10^−8^
	Mixed edible tissues	1.27 × 10^−4^		1.27 × 10^−1^		4.83 × 10^−8^
Male	Intestine and Hepatopancreas	2.14 × 10^−3^		2.14 × 10^0^		8.13 × 10^−7^
	Muscle	2.91 × 10^−6^		2.91 × 10^−3^		1.11 × 10^−9^
	Heart	5.83 × 10^−5^		5.83 × 10^−2^		2.22 × 10^−8^
	Mixed edible tissues	3.33 × 10^−4^		3.33 × 10^−1^		1.27 × 10^−7^

**Table 7:** Cadmium concentrations in sediments from different regions of China.

Sample Sites	Cd (mg/kg)	References
Bohai bay	0.22	[42]
Xiamen, East China Sea	1.74–17.2	[43]
Yellow river	0.27–1.43	[44]
Daya bay	18.68–89.58	[45]
Yellow River Estuary	0.14	[46]
Pearl River Delta	1.04	[47]
Yangtze River Estuary adjacent waters	0.67 ± 0.25	[48]
Hangzhou Bay	0.11	[49]
Near Sino-Singapore Tianjin Eco-City	0.17	[50]
Guangxi Beibu Gulf coastal zone	0.088	[51]
The Jialu River	2.12–3.64	[52]
The Fenghe River Basin	0.18–0.48	[53]

## Data Availability

The original contributions presented in the study are included in the article, further inquiries can be directed to the corresponding author.
